# Second-line treatment with irinotecan plus cisplatin *vs* cisplatin of patients with advanced non-small-cell lung cancer pretreated with taxanes and gemcitabine: a multicenter randomised phase II study

**DOI:** 10.1038/sj.bjc.6602748

**Published:** 2005-09-20

**Authors:** V Georgoulias, A Agelidou, K Syrigos, A Rapti, M Agelidou, J Nikolakopoulos, A Polyzos, A Athanasiadis, E Tselepatiotis, N Androulakis, K Kalbakis, G Samonis, D Mavroudis

**Affiliations:** 1 Department of Medical Oncology, University General Hospital of Heraklion, PO Box 1352, 711 10 Heraklion, Crete, Greece; 2 1st Department of Pulmonary Disease, ‘Sotiria’ General Hospital, Athens, Greece; 3 Medical Oncology Unit, 3rd University Department of Medicine, ‘Sotiria’ General Hospital, Athens, Greece; 4 8th Department of Pulmonary Diseases, ‘Sotiria’ General Hospital, Athens, Greece; 5 2nd Department of Pulmonary Diseases, ‘Sismanoglion’ General Hospital of Athens, Athens, Greece; 6 1st Department of Pulmonary Diseases, ‘Sismanoglion’ General Hospital of Athens, Athens, Greece; 7 Medical Oncology Unit, University Department of Propedeutic Medicine, ‘Laikon’ General Hospital of Athens, Athens, Greece; 8 Department of Medical Oncology, General Hospital of Larissa, Larissa, Greece; 9 Department of Internal Medicine, ‘Patision’ General Hospital of Athens, Athens, Greece

**Keywords:** irinotecan, cisplatin, second-line treatment, NSCLC

## Abstract

The aim of this study was to compare the irinotecan/cisplatin regimen with cisplatin as second-line chemotherapy in patients with advanced non-small-cell lung cancer (NSCLC) pretreated with a taxane/gemcitabine regimen. Patients (*n*=147) with stage IV NSCLC pretreated with a taxane/gemcitabine regimen were randomly assigned to receive either irinotecan (110 mg m^−2^, day 1 and 100 mg m^−2^, day 8) and cisplatin (80 mg m^−2^, day 8) (IC; *n*=74) or CDDP (80 mg m^−2^, day 1) (C; *n*=73) every 3 weeks. Patients treated with IC and C had a median survival of 7.8 and 8.8 months, respectively (*P*=0.933). The 1-year survival rate was 34.3% for IC-treated patients and 31.7% for C-treated patients. Cox's regression analysis revealed that response to treatment (hazard ratio (HR)=2.787; 95% confidence interval (CI): 1.1578–4.922) and performance status (HR=1.865; 95% CI: 1.199–2.872) was independent prognostic factors for survival. Overall response rate was 22.5% (95% CI: 12.8–32.2%) for IC-treated patients and 7.0% (95% CI: 1.15–13.6%) for C-treated patients (*P*=0.012); tumour growth control (partial remission (PR)+stable disease (SD)) was observed in 26 (38%) IC and 25 (36%) C patients (*P*=0.878). There was no difference in terms of quality of life between the two chemotherapy arms. The incidence of febrile neutropenia, grade 3 and 4 neutropenia and grade 3 and 4 diarrhoea was significantly higher in the IC- than the C-treated patients. Other toxicities were mild. There were no treatment-related deaths in either arm. The IC regimen did not confer a survival benefit compared with C as second-line treatment of patients with advanced NSCLC pretreated with a taxane/gemcitabine regimen, despite its better efficacy in terms of response rate.

The use of front-line chemotherapy in the treatment of advanced non-small-cell lung cancer (NSCLC) has been expanded as a result of its increased use in the context of multimodality treatment for stage IIIA and IIIB disease (Belani, 1993; Lilenbaum and Green, 1993; Bunn and Kelly, 1998; Webb and O’Brien, 1998) and the development of new active drugs in both chemotherapy-naïve and pretreated patients (Fossella *et al*, 1997; Androulakis *et al*, 1998; Ferrigno and Buccheri, 2000; Huisman *et al*, 2000; Iaffaiolli *et al*, 2000). This increased the interest in second-line chemotherapy for good performance status (PS) patients with NSCLC. Indeed, two randomised studies clearly demonstrated that second-line treatment with docetaxel conferred a statistically significant survival benefit, improved quality of life and clinical benefit over either best supportive care (Shepherd *et al*, 2000) or monotherapy with either vinorelbine or ifosfamide (Fossella *et al*, 2000). In addition, premetrexed administration in the second-line setting in patients with NSCLC resulted in equivalent efficacy compared with docetaxel but with a better toxicity profile (Hanna *et al*, 2004). Moreover, the tyrosine kinase inhibitor of the epidermal growth factor receptor, erlotinib (Tarceva), showed a progression-free and overall survival benefit over placebo when it was administered as second- or third-line treatment (Shepherd *et al*, 2004).

Since platinum-based chemotherapy still remains the most commonly used standard of care for patients with advanced NSCLC, the majority of studies of second-line chemotherapy have targeted a patient population that has received front-line platinum-based chemotherapy. However, front-line nonplatinum-based regimens, with their favourable toxicity profile, show similar activity in terms of overall response rate, response duration, time to tumour progression (TTP) and overall survival as platinum-based combinations (Yamamoto *et al*, 2000; Chen *et al*, 2001; Georgoulias *et al*, 2001, 2005; Giaccone *et al*, 2002; Gridelli *et al*, 2002; Kosmidis *et al*, 2002). Therefore, the number of patients treated with nonplatinum combinations in the first-line setting is increasing, and it would be of interest to develop salvage chemotherapy regimens for this particular group of patients.

Irinotecan (CPT-11) is a semisynthetic derivative of the plant alkaloid, camptothecin. Irinotecan appears to exert its antineoplastic activity via the inhibition of the nuclear enzyme topoisomerase I, and phase II studies have shown that the drug is active in NSCLC (Boisseau *et al*, 1996; O'Reilly and Rowinski, 1996). An additive or synergistic effect for the combination of irinotecan and cisplatin (CDDP) has also been described (Kano *et al*, 1992; Kudoh *et al*, 1993). Early phase I and II studies demonstrated that the combination of irinotecan and cisplatin is active in chemotherapy-naïve patients with NSCLC, achieving response rates up to 50% (Masuda *et al*, 1992, 1993; Mori *et al*, 1997; Ueoka *et al*, 1999; Wagner *et al*, 1999; Kakolyris *et al*, 2000). Moreover, the combination of irinotecan and cisplatin resulted in an objective response rate (ORR) ranging from 22 to 29% and a median survival time of 8 months in both platinum-refractory patients with advanced NSCLC (Nakanishi *et al*, 1999) and patients who were previously treated with a taxane/gemcitabine regimen (Kakolyris *et al*, 2001). The main toxicities were severe neutropenia, diarrhoea and fatigue (Kakolyris *et al*, 2001).

Based on the promising activity of irinotecan/cisplatin combination in our phase I (Kakolyris *et al*, 2000) and II (Kakolyris *et al*, 2001) studies, we decided to conduct a prospective, multicentre, randomised phase III study to compare the efficacy and tolerance of this regimen *vs* single agent cisplatin in platinum-naïve patients with advanced NSCLC pretreated with a taxane/gemcitabine regimen.

## PATIENTS AND METHODS

### Patients

Patients (aged >18 years) with a World Health Organisation (WHO) PS of 0–2 and histologically or cytologically confirmed stage IIIB or IV NSCLC were enrolled into this trial. Additional inclusion criteria were as follows: prior chemotherapy with a taxane/gemcitabine-based regimen in the first-line setting; at least one bidimensionally measurable lesion outside an irradiation field; absence of a second primary tumour, except for basal cell carcinoma of the skin or carcinoma *in situ* of the cervix; adequate bone marrow, kidney and liver functions (with the exception of alkaline phosphatase, which could be up to five times the UNL in case of liver metastases); and a negative pregnancy test in women of childbearing age. Prior radiotherapy was allowed, provided that it had been completed at least 4 weeks prior to enrolment and ⩽25% of the total bone marrow had been irradiated. At least 4 weeks had to have elapsed from completion of the last cycle of front-line chemotherapy. Patients were excluded if they had clinically uncontrolled brain metastases or peripheral neuropathy greater than WHO grade 1. Other exclusion criteria were as follows: severe cardiopulmonary insufficiency, severe angina pectoris or myocardial infraction within 6 months prior to study entry, active infection, severe malnutrition (loss of >15% of body weight) or a life expectancy of <3 months. The trial has been approved by the Ethics and Scientific Committees of the participating Institutions and all patients had to sign written informed consent in order to participate in the study.

### Treatment plan and dose modifications

Patients were centrally registered and eligible patients were stratified according to their PS and the stage of the disease. Patients were randomised to receive either irinotecan (Campo; CPT-11, Aventis Pharma, Antony, France) at the dose of 110 and 100 mg m^−2^ on days 1 and 8 (Kakolyris *et al*, 2000, 2001), respectively, and cisplatin (Platinol; CDDP, Bristol Meyers Squibb, Princeton, NJ, USA) at the dose of 80 mg m^−2^ on day 8 (IC arm) or cisplatin at the same dose on day 1 (C arm). Standard hydration and forced diuresis were used for the administration of cisplatin. In both arms, cycles were repeated every 3 weeks. Three chemotherapy cycles were administered followed by three additional cycles in case of objective response or stable disease; treatment was discontinued in case of progressive disease or intolerable toxicity. All patients received standard antiemetic therapy with odansteron 16 mg and dexamethasone 8 mg given intravenously (i.v.) 30 min prior to chemotherapy administration. Loperamide was used for the treatment of delayed diarrhoea due to irinotecan, according to the manufacturer's instructions.

Dose modifications were performed according to the haematologic toxicity and diarrhoea. Patients developing grade 3 and 4 neutropenia without fever received the subsequent cycles with prophylactic recombinant human/granulocyte colony-stimulating factor (rhG-CSF: Granocyte, Aventis Pharma) at the dose of 150 *μ*g m^−2^ from day 9 to day 15, in order to maintain a reasonable dose intensity. In case of persistent neutropenia despite the prophylactic use of rhG-CSF administration or in case of febrile neutropenia (fever more than 37.5°C for at least 24 h), the day 8 doses of both drugs were reduced by 25% in all subsequent cycles. In case of grade 3 or 4 delayed diarrhoea, all subsequent cycles were administered with a 25% reduction of the irinotecan dose. Patients requiring more than one dose reduction were withdrawn from the study.

### Baseline and follow-up assessments

Baseline assessments included complete medical history and physical examination, complete blood cell count with differential and serum chemistry. Bidimensionally measurable disease was determined by standard imaging procedures at baseline (chest X-ray, CT scans of the thorax, abdomen and brain and whole-body bone scan). Abdominal ultrasonography and magnetic resonance imaging scans were performed if indicated. Follow-up brain CT scans and liver or adrenal ultrasound exams were performed at the discretion of the treating physician. Tumour assessment for response was performed every three chemotherapy cycles. Complete medical history and physical examination, as well as complete blood cell count with differential and serum chemistry were performed every 3 weeks. Treatment-related haematologic toxicity was evaluated weekly, and daily in patients with grade 3 and 4 neutropenia, febrile neutropenia or thrombocytopenia.

For the quality of life assessment, the Lung Cancer Symptom Scale (LCSS) and the EuroQOL (EQ-5D) questionnaire were used at baseline and every three chemotherapy cycles thereafter (Hollen *et al*, 1993; Rabin and de Charro, 2001).

Patients who received at least three cycles of chemotherapy were assessed for response according to WHO criteria (World Health Organization, 1979). All responses had to be maintained for at least 4 weeks and were confirmed by an independent panel of radiologists. Patients who received at least one chemotherapy cycle were assessed for toxicity. The standard WHO criteria were used for the evaluation of toxicity (World Health Organization, 1979).

### Statistical considerations

This was a prospective, multicentre, randomised phase II trial. The primary end point was the comparison of median survival times. Secondary end points included objective tumour response rates, duration of response, TTP, treatment tolerance and quality of life. For the sample size calculation, the primary outcome measure was survival time: 8 months for the IC group and 4 months for C group. In all, 65 patients/arm were required for the study to demonstrate a significant difference (at the 5% level) between the two survival curves with a power of 90%.

Differences of rates between groups were assessed by Pearson's *χ*^2^ test or Fisher's test where appropriate. Time-to-event end points were calculated using Kaplan–Meier methods with appropriate censoring. The independent influence of several factors on the risk of nonresponse, relapse or nonsurvival was assessed by logistic regression, while that on the hazards of relapse or failure of survival by Cox's proportional-hazards model (Collet, 1999; Tangent and Koch, 1999). Survival was calculated from the date of randomisation until the date of death. TTP was assessed from the date of randomisation until the date of disease progression. Response duration was calculated from the date that the criteria of response were met for the first time until the date of documentation of disease progression.

## RESULTS

### Patients’ demographics

From August 1999 to August 2002, 147 pretreated patients with NSCLC were registered and randomised to receive either IC (*n*=74) or C (*n*=73). Most of the patients (85%) had received front-line chemotherapy with docetaxel and gemcitabine in the context clinical trials conducted by the Hellenic Oncology Research Group (HORG); 15% of the patients had received a combination of paclitaxel/gemcitabine in the first-line setting. Three patients in IC (one did not fulfil the inclusion criteria and two had never received treatment) and five patients in C (one had a second primary tumour and four had never received treatment) were not evaluable. Table 1 demonstrates patient characteristics. The median age was 64 and 68 years for IC and C, respectively. The two groups were well balanced with respect to gender, PS, histology and extension of the disease (with a median number of two involved organs/patient) as well as the pretreatment disease parameters. All patients in both arms had stage IV disease. The median interval time from the last chemotherapy cycle was 1.4 (range, 1–27.7) and 2.9 months (range, 1–54) in the IC and C arms, respectively; in addition, 52 (73%) and 35 (53%) patients enrolled in the IC and C arms, respectively, had resistant or refractory disease. In all, 20 (28%) and 13 (19%) of IC- and C-treated patients, respectively, received third-line chemotherapy, which mainly consisted of single agent vinorelbine (*n*=8 patients in both groups), gemcitabine (*n*=10 patients), gefitinib (*n*=5 patients), topotecan (*n*=5 patients) or other regimens off protocol.

### Survival

**Figure 1:**
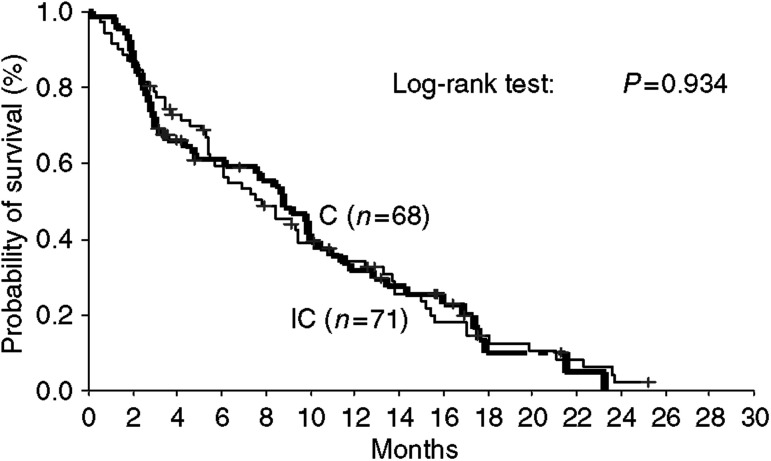
Kaplan–Meier survival curves of patients treated with IC and C regimens.

The median survival times were 7.8. (range, 0.5–25.2) and 8.8 (range, 0.5–23.3) months in patients treated with IC and C, respectively (log-rank test: *P*=0.934) (Figure 1). The 1-year survival rates were 34.3 and 31.7% in IC and C arms, respectively. Survival was not affected by age, gender, histology and the number of tumour sites involved. Conversely, responders to second-line treatment had significantly better survival time (median 17.0 months; range, 4–25.2) than those who failed to respond (median 7.6 months; range, 0.5–23.8) (*P*=0.0001). Moreover, the survival time was significantly better in patients with PS of 0–1 (median 9.4 months; range, 0.5–25.2) than those with PS of 2 (median 4.5 months; range, 1.0–22.3) (*P*=0.006). Both effects were independent of the regimen used. Cox's regression analysis confirmed that these two factors had an independent effect on the hazard of death. The risk of death for nonresponders to chemotherapy was about three times higher than that of responders (hazard ratio (HR)=2.787; 95% confidence interval (CI) 1.578–4.922); similarly, the risk of death of patients with a PS of 2 was about two times higher than that of patients with PS of 0–1 (HR=1.865, 95% CI 1.199–2.872) (*P*=0.005).

The median follow-up period was 7.0 (range, 0.5–25) and 6.5 (range, 0.5–23) months for IC and C patients, respectively (*P*=0.580). During this period, 61 (85.9%) IC-treated patients and 52 (76.5%) C-treated patients died (*P*=0.153). In the IC arm, the causes of death were as follows: disease progression (*n*=58); lower respiratory infection and septicaemia leading to acute renal failure (*n*=1); cardiorespiratory failure (*n*=1); and ventricular arrhythmia probably due to myocardial ischaemia (*n*=1). In the C arm, the reason of death was disease progression (*n*=52).

### Response to treatment

Seven patients in the IC and six in the C arm were lost to follow-up and were considered as progressors in the intention-to-treat analysis. There were no complete responses in either chemotherapy arm. In all, 16 (22.5%; 95% CI: 12.8–32.2%) patients in IC arm and five (7%; 95% CI: 1.15–13.6%) in C arm had a partial response (*P*=0.012). Stable disease was observed in 11 (15.5%) and progressive disease in 44 (62%) IC patients; similarly, stable and progressive disease was documented in 20 (29%) and 43 (63%) patients in the C arm, respectively. The tumour growth control rate (complete remission (CR)+partial remission (PR)+stable disease (SD)) was 38% in the IC arm and 36% in the C arm (Fisher's exact test; *P*=0.878). In all, 11 (20%) IC-treated patients and two (4%) C-treated patients who responded to second-line chemotherapy had failed to respond to front-line chemotherapy; moreover, the IC regimen resulted in an almost three-fold higher incidence of objective responses compared with the C regimen in patients with sensitive (ORR: 21 *vs* 6%, respectively; *P*=0.113) or resistant/refractory (23 *vs* 8%, respectively; *P*=0.071) disease. The incidence of objectively validated response in lesions located in the lung or lymph nodes was significantly higher in patients treated with IC than with C (ORR: 23 *vs* 8%, *P*=0.012 for lung and ORR: 28 *vs* 7%, *P*=0.012 for lymph nodes); there was no difference in the ORRs for lesions located in the liver (13 *vs* 9%; *P*=0.738), pleura (27 *vs* 13%; 0=0197) or adrenal (14 *vs* 0%; *P*=0.242). Response was significantly affected by PS, since 15 (26%) and three (6%) patients with PS of 0 and 1 treated with IC and C, respectively, achieved an objective response (*P*=0.004). The risk of nonresponse for patients with PS 0 and 1 in group C was almost six times higher than that of patients with the same PS in the IC group (odds ratio (OR): 5.814; 95% CI: 1.577–21.438). The treatment regimen was revealed to be the only independent predictive factor for response (*P*=0.017) (OR=3.665; 95% CI: 1.261–10.656). The median duration of response was 6 months (range, 2.1–17.8) in IC-treated patients and 12 months (range, 2.3–12.2) in C-treated patients (log-rank test: *P*=0.154). The median TTP was 2.6 (range, 1–20.3) and 2.1 (range, 1–17.6) months in the IC and C arms, respectively (log-rank test; *P*=0.641); the 1-year progression-free survival rates were 6.4 and 10.9% in the IC and C arms, respectively.

### Compliance with the treatment

A total of 247 IC (median three cycles/patient (range, 1–9)) and 243 C cycles (median three cycles/patient (range, 1–6)) were administered. The median interval between cycles was 23 days (range, 21–37) in the IC and 21 days (range, 21–27) in C arm. The median dose intensity for patients randomised to IC was 56 mg m^−2^ week^−1^ (range, 29–70) for irinotecan and 22 mg m^−2^ week^−1^ (range 9–27) for cisplatin corresponding to 80 and 81% of the protocol planned doses, respectively. The median dose intensity of cisplatin in patients randomised to arm C was 26.6 mg m^−2^ week^−1^ (range, 19–27), which corresponded to 99% of the planned dose.

In all, 77 (31%) and 30 (12%) cycles were delayed in IC and C arms, respectively (*P*=0.0001). A total of 49 (20%) IC and 10 (4%) C cycles were delayed more than 7 days (*P*=0.005). The reasons for treatment delay were as follows: haematological toxicity (IC arm: *n*=24 cycles; C arm: *n*=8 cycles; *P*=0.004); nonhaematological toxicity (IC arm: *n*=12 cycles; C arm: *n*=1 cycle; *P*=0.002); both haematologic and nonhaematologic (IC arm: *n*=1 cycle; C arm: *n*=0 cycles); and finally, for reasons unrelated to treatment or disease (40 IC and 21 C cycles).

In all, 52 (21%) IC and four (2%) C cycles, respectively, required dose reductions (*P*=0.0001). The reasons for dose reduction were as follows: haematological toxicity (IC arm: *n*=11 cycles; C arm: *n*=0 cycles); nonhaematological toxicity (IC arm: *n*=19 cycles; C arm: *n*=3 cycles); both haematological and nonhaematological toxicity (IC arm: *n*=4 cycles; C arm: *n*=0 cycles); and for reasons unrelated to disease or treatment (IC arm: *n*=16 cycles; C arm: *n*=1 cycle).

### Toxicity

The haematological and nonhaematological toxicities are summarised in Table 2. Grade 3 and 4 neutropenia occurred in 22 (31%) and three (4%) patients in IC and C arms, respectively (*P*=0.001). A total of 128 (52%) and 94 (39%) cycles in IC and C, respectively, required prophylactic rhG-CSF support (*P*=0.001). Five (7.0%) patients treated with IC developed febrile neutropenia. All were hospitalised and uneventfully recovered following treatment with i.v. broad-spectrum antibiotics and rhG-CSF support. There was no case of febrile neutropenia in patients treated with C. Grade 2–4 anaemia was observed in 27 (38%) IC and 20 (30%) C patients (*P*=0.283). Five (7%) patients in the IC and two (3%) patients in the C arm developed grade 3 and 4 thrombocytopenia (*P*=0.269). No patient developed bleeding episodes requiring platelet transfusions or hospitalisation.

The nonhaematological toxicity was relatively mild. Grade 3 and 4 nausea/vomiting was reported by nine (13%) IC and three (4%) C patients. There was a significantly higher incidence of grade 3 and 4 diarrhoea in patients treated with IC (*n*=19; 27%) than in those treated with C (*n*=2; 4%) (*P*=0.0001); hospitalisation was required in 15 out of 19 IC (median duration of hospitalisation: 6 days) and in two out of two C patients with grade 3 and 4 diarrhoea. All patients recovered. Grade 2–4 asthenia was reported by 21 (30%) IC and 21 (31%) C patients. Other toxicities were mild. The toxicity profile of IC and C was not modified by patients’ PS. Grade 3 and 4 neutropenia and diarrhoea remained the most serious adverse events associated with the IC regimen in patients with either PS of 0–1 or 2 (Table 3).

### Symptom and quality of life assessment

Patients’ compliance with quality of life assessment for IC group was 98% at baseline, 73% at the third cycle and decreased to 31% at the end of chemotherapy (EoC). Similarly for the C group, compliance was 100, 75 and 30%, respectively. Disease-related symptoms revealed no significant differences between the two arms at baseline and during treatment (at third cycle and EoC).

Patients treated with IC reported no significant differences between baseline and EoC assessment. Similarly, no significant differences were observed for C group, while a trend towards improvement was observed for cough (*P*=0.069).

## DISCUSSION

The present multicentre, randomised phase II study was designed in order to evaluate the role of platinum-based chemotherapy as second-line treatment in patients with advanced NSCLC who have received first-line chemotherapy with a taxane/gemcitabine regimen. The results demonstrate that although the combination of irinotecan/cisplatin resulted in a significantly higher response rate than cisplatin monotherapy, there was no difference between the two chemotherapy arms in terms of 1-year survival, median overall survival, duration of response and TTP. However, it should be stressed that our findings should not be generalised for patients treated with other than taxane/gemcitabine nonplatinum-containing regimens in the first-line setting.

There are very few data in the literature concerning the role of platinum compounds in the second-line setting in patients with advanced NSCLC. This may be due to the consideration of platinum-based combinations as ‘gold standard treatment’ for the front-line setting; moreover, the cumulative neurotoxicity of cisplatin may preclude further treatment with this agent after failure of a front-line platinum-based regimen. In the present study, we demonstrated that second-line treatment with either irinotecan (CPT-11) plus cisplatin (IC) or cisplatin (C) alone in patients pretreated with taxanes and gemcitabine could confer a similar overall median survival (7.8 and 8.8 months, respectively). This should be attributed to the fact that both regimens resulted in a similar tumour growth control rate (38 and 36% for IC and C regimens, respectively). The Cox regression analysis demonstrated that response to chemotherapy (CR+PR) and PS (0–1) were independent prognostic factors for survival, irrespectively of the chemotherapy regimen. It is interesting to note that second-line cisplatin resulted in a median overall survival of 8.8 months, while second-line docetaxel and premetrexed in 7.5 and 8.3 months, respectively (Fossella *et al*, 2000; Shepherd *et al*, 2000; Hanna *et al*, 2004).

The low antitumour activity (ORR=7%) of CDDP in our patients is in agreement with previous reports demonstrating responses of less than 10% (Belani, 1998). Conversely, the irinotecan/cisplatin combination resulted in a significantly higher ORR (=22.5%) confirming our previous observation (Kakolyris *et al*, 2001). Similarly, a 31% response rate was observed (Nakanishi *et al*, 1999) with a weekly administration of irinotecan and cisplatin in 16 patients with refractory NSCLC. A recent multicenter, randomised phase II study, which compared the irinotecan/gemcitabine combination *vs* irinotecan in NSCLC patients pretreated with taxanes plus cisplatinum, demonstrated a poor antitumour activity of irinotecan (ORR=4.2%) (Georgoulias *et al*, 2004) as already reported by Negoro *et al* (see Ferrigno and Buccheri, 2000). Taken together, the improved antitumour activity of irinotecan/cisplatin combination (Nakanishi *et al*, 1999; Kakolyris *et al*, 2001) and the poor activity of single agent irinotecan or cisplatin in the second-line setting seem to indicate an *in vivo* synergism between the two agents as it has been shown in preclinical studies (Kano *et al*, 1992; Kudoh *et al*, 1993).

The irinotecan/cisplatin regimen was active in patients failing to respond to front-line taxane/gemcitabine-based regimens. Moreover, this combination was equally active in both sensitive and resistant/refractory to taxane/gemcitabine tumours. As the chemosensitivity to first-line chemotherapy may influence the results with second-line chemotherapy, one should consider that the tumour growth control rates achieved with front-line treatment were different for the two arms: 49% for the IC *vs* 72% for the C arm. This imbalance has to be attributed to a selection bias of the randomisation procedure since the patients were not stratified according to their response to previous front-line chemotherapy. However, logistic regression analysis revealed that only the chemotherapy regimen was an independent predictive factor for response. Similar results have been previously reported in phase II studies (Kakolyris *et al*, 2001). Several phase II studies have also shown that platinum-based chemotherapy regimens may be active in the second-line setting (Gridelli *et al*, 1992; Stathopoulos *et al*, 1999; Huisman *et al*, 2001; De Pas *et al*, 2001). It is interesting to note that despite the higher response rate achieved with IC, there was no difference in terms of quality of life between IC and C. We do not have a clear explanation for this observation, although we cannot exclude that it may be due to the similar tumour growth control rate achieved by the two regimens.

Second-line chemotherapy in advanced NSCLC is a palliative treatment. Therefore, chemotherapy regimens used in this setting should lack severe toxicity in the interest of patients’ quality of life. The chemotherapy regimens used in the present study displayed a manageable toxicity profile and there was no treatment-related death. However, the irinotecan/cisplatin regimen was associated with a significantly higher incidence of grade 3 and 4 neutropenia and neutropenic fever than cisplatin monotherapy, leading to a higher proportion of patients requiring prophylactic use of G-CSF. The IC regimen was also associated with a higher incidence of grade 3 and 4 diarrhoea than single agent cisplatin; this adverse event necessitated patients’ hospitalisation for administration of i.v. broad-spectrum antibiotics and hydration. Although a pharmacoeconomic evaluation of the two regimens was not performed, it is obvious that these two main toxicities of the IC regimen required patient hospitalisation, which is not without economic consequences. This is an important issue, especially when taking into account the similar overall survival achieved by the two chemotherapy regimens and the more favourable toxicity profile of cisplatin monotherapy.

In conclusion, our observations indicate that second-line CDDP is equally effective in terms of overall survival, TTP and quality of life as the irinotecan/CDDP regimen in patients with advanced NSCLC pretreated with a taxane/gemcitabine front-line combination. This finding taken together with our prior observation that single agent irinotecan given in the second-line setting resulted in a similar overall survival of patients with NSCLC as the irinotecan/gemcitabine combination (Agelaki *et al*, 2001) raise the question of whether monotherapy is, indeed, a sufficient second-line treatment of NSCLC. However, additional studies evaluating different doses and/or administration schedules as well as novel agent combinations are needed to further elucidate the value of second-line chemotherapy in patients with advanced NSCLC.

## Figures and Tables

**Table 1:** Patient characteristics

	**IC group**		**C group**	
	***n***	**%**	***n***	**%**
Patients enrolled	74		73	
Patients eligible and evaluable for response and toxicity	71	96	68	93
*Age (years)*				
Median (range)	61 (36–77)		64 (36–78)	
				
*Gender*				
Male	64	90	58	85
Female	7	10	10	15
				
*Stage*				
III	—	—	—	—
IV	74	100	73	100
				
*PS (WHO)*				
0–1	58	82	53	78
2	13	18	15	22
				
*Histology*				
Squamous	26	37	24	35
Adenocarcinoma	33	47	33	49
Large cell	1	1	1	1
Undifferentiated	11	15	10	15
				
*Prior treatment*				
Surgery	13	18	9	13
Radiotherapy (adjuvant/curative)	12	17	11	16
Chemotherapy	74	100	68	100
				
*Line of chemotherapy*				
Second	68	96	61	90
Third	3	4	7	10
				
*Objective response to first-line chemotherapy*				
CR	—	—	1	2
PR	17	24	22	32
SD	18	25	26	38
PD	36	51	19	28

PS=performance status; WHO=World Health Organisation; CR=complete remission; PR=partial remission; SD=stable disease; PD=progressive disease.

**Table 2:** Haematologic and nonhaematologic toxicity of second-line IC and C

	**Grade 1**		**Grade 2**		**Grade 3**		**Grade 4**	
	**IC**	**C**	**IC**	**C**	**IC**	**C**	**IC**	**C**
Anaemia	38 (54)	35 (52)	23 (32)	19 (28)	4 (6)	—	—	1 (2)^a^
Neutropenia	11 (16)	9 (13)	13 (18)	7 (10)	12 (17)	3 (4)	10 (14)^b^	—
Thrombocytopenia	18 (25)	12 (18)	3 (4)	1 (2)	3 (4)	2 (3)	2 (3)	—
Nausea/vomiting	11 (16)	4 (6)	18 (25)	13 (19)	8 (11)	3 (4)	1 (1)^c^	—
Diarrhoea	8 (11)	3 (4)	16 (23)	2 (3)	14 (20)	1 (2)	5 (7)^d^	1 (2)
Mucositis	2 (3)	2 (3)	—	—	—	—	—	1 (2)
Neurotoxicity	2 (3)	4 (6)	3 (4)	3 (4)	—	1 (2)	—	—
Asthenia	19 (27)	8 (12)	13 (18)	12 (18)	8 (11)	7 (10)	—	2 (3)
Fluid retention syndrome	2 (3)	—	2 (3)	1 (2)	—	1 (2)	1 (1)	1 (2)
Non-neutropenic infection	9 (13)	6 (9)	—	—	—	—	—	1 (2)

The results are expressed as the ‘number of patients’. In parentheses: % of patients.

a Grade 2–4 anaemia; *P*=0.283.

b Grade 3 and 4 neutropenia; *P*=0.001.

c Grade 3 and 4 nausea/vomiting; *P*=0.083.

d Grade 3 and 4 diarrhoea; *P*=0.0001.

**Table 3:** Grade 3 and 4 haematologic and nonhaematologic toxicities according to PS

	**PS 0 and 1**			**PS 2**		
**Toxicity**	**CPT-11/CDDP (*n*=56)**	**CDDP (*n*=52)**	***P*-value**	**CPT-11/CDDP (*n*=15)**	**CDDP (*n*=16)**	***P*-value**
Anaemia^a^	17 (30.3%)	14 (26.9%)	0.693	10 (66.7%)	6 (37.6%)	0.104
Neutropenia	15 (26.8%)	3 (5.8%)	0.011	9 (59.7)	—	0.0001
Thrombocytopenia	3 (5.4%)	1 (2.0%)	0.345	2 (13%)	1 (6.3%)	0.505
Febrile neutropenia	4 (7.2%)	—	0.169	1 (6.7%)	—	0.294
Nausea/vomiting	5 (9.0%)	1 (2.0%)	0.112	4 (26.7%)	2 (12.5%)	0.318
Diarrhoea	12 (21.6%)	—	0.0001	7 (46.7%)	2 (12.5%)	0.036
Asthenia^a^	13 (23.2%)	16 (30.7%)	0.202	8 (53%)	5 (31.4%)	0.372

PS=performance status.

a Grade 2–4.

## Appendix A1

Participating centres and investigators
